# Occluded object detection and exposure in cluttered environments with automated hyperspectral anomaly detection

**DOI:** 10.3389/frobt.2022.982131

**Published:** 2022-10-14

**Authors:** Nathaniel Hanson, Gary Lvov, Taşkın Padir

**Affiliations:** ^1^ Institute for Experiential Robotics, Northeastern University, Boston, MA, United States; ^2^ Department of Electrical and Computer Engineering, Northeastern University, Boston, MA, United States

**Keywords:** cluttered environment, hyperspectral imaging, multi-modal scene segmentation, system architecture, automated machine learning

## Abstract

Cluttered environments with partial object occlusions pose significant challenges to robot manipulation. In settings composed of one dominant object type and various undesirable contaminants, occlusions make it difficult to both recognize and isolate undesirable objects. Spatial features alone are not always sufficiently distinct to reliably identify anomalies under multiple layers of clutter, with only a fractional part of the object exposed. We create a multi-modal data representation of cluttered object scenes pairing depth data with a registered hyperspectral data cube. Hyperspectral imaging provides pixel-wise Visible Near-Infrared (VNIR) reflectance spectral curves which are invariant in similar material types. Spectral reflectance data is grounded in the chemical-physical properties of an object, making spectral curves an excellent modality to differentiate inter-class material types. Our approach proposes a new automated method to perform hyperspectral anomaly detection in cluttered workspaces with the goal of improving robot manipulation. We first assume the dominance of a single material class, and coarsely identify the dominant, non-anomalous class. Next these labels are used to train an unsupervised autoencoder to identify anomalous pixels through reconstruction error. To tie our anomaly detection to robot actions, we then apply a set of heuristically-evaluated motion primitives to perturb and further expose local areas containing anomalies. The utility of this approach is demonstrated in numerous cluttered environments including organic and inorganic materials. In each of our four constructed scenarios, our proposed anomaly detection method is able to consistently increase the exposed surface area of anomalies. Our work advances robot perception for cluttered environments by incorporating multi-modal anomaly detection aided by hyperspectral sensing into detecting fractional object presence without need for laboriously curated labels.

## 1 Introduction

In environments filled with unorganized objects, detecting items that do not belong is a significant problem in robot manipulation with numerous applications. For example, in food processing, it is advantageous to detect foreign objects from conveyor belts full of items that are safe for consumption. Within a domestic setting, a similar problem class arises within the mundane task of doing laundry; separating machine washable clothes from those that are not. Such tasks are monotonous and repetitive, thus making them perfect candidates for robots. Prior work has focused on specialized object classification models to address this problem Barnabé et al. (2015); Erickson et al. (2019); Ku et al. (2021); Kwak et al. (2021). However, application specific solutions can become resource intensive in data acquisition, labeling, and tuning. A generalized approach has the potential to significantly reduce the time needed to train and deploy new machine learning models.

Automation research is commonly predicated on scenarios where robots are presented with easily separable items. The assumption of separability aids in the demonstration of other capabilities like grasp selection; nonetheless, it may not necessarily be indicative of environments encountered in the field. Real world environments rarely reflect an easily separable ordering, where objects can be easily segmented without any occlusions. Such organization presupposes an unrealistic tendency towards order, instead of disorder. To become pervasive assistant, robots must learn to contend with the clutter.

Prior approaches to anomaly detection within clutter have focused on using standard Red Blue Green (RGB) camera images to segment scenes Zeng et al. (2018a); Zeng et al. (2018b); Bejjani et al. (2019). However, a generalized solution would ideally be able to effectively differentiate between objects that are similar in appearance but different in underlying chemical-physical properties. At a glance, a slightly spoiled fruit may look the same as a ripe one. Discerning such visually minute differences solely from RGB data would be insufficient. Another potential drawback of RGB data is its lack of robustness to lighting changes. This deficiency can be further exacerbated by optical effects such as specular reflections or shadows. Furthermore, state of the art methods that use Convolutional Neural Networks (CNN) for anomaly detection require thousands Shahinfar et al. (2020) of manually labeled images to form appropriately sized training sets. Image augmentation Shorten and Khoshgoftaar (2019) may be used to reduce the amount of images required for training and testing a deep network, but these supervised solutions require the intervention of a human-expert to tailor labels to the present scenario, which can be impractical.

To simultaneously address both the weaknesses of RGB data and to circumvent the intensive process of creating a labeled dataset, we leveraged Hyperspectral Imaging (HSI). HSI captures additional wavelength information about the environment, with the size of data obtained from the sensor being orders larger than RGB data. HSI outputs a three-dimensional datacube with reflectance measurements at multiple wavelengths of light spanning the Visible to the Near Infrared (VNIR) spectrum. Each pixel in the hyperspectral datacube, has multiple discrete wavelength values, indicating what proportion of light was reflected at that specific wavelength. Across the spectrum, these values vary as a function of the material type. These spectral signatures involve no contact with the objects, and are able to effectively characterize objects that to RGB cameras appear indistinguishable.

In this work, we first fuse RGB-Depth data with HSI data. By interpreting this data with clustering methods, we are able to autonomously create a binary classification map where the two labels are objects that belong to the pure class and objects that are anomalous. This process is conducted with the assumption that there is a preponderance of one desired material. Using this binary classification map, a coarse initial classification is employed to train an autoencoder (AE) for locating anomalous regions. Our results show that a simple clustering model is sufficient to train the relatively complex AE model, with the AE being able to more effectively classify anomalies than the simple cluster approach. Our framework requires no prior knowledge of the scene other than an assumption of a majority of one desired material.

Since the RGB and hyperspectral data are annotated with depth information, this facilitates the determination of the cartesian coordinates of potentially anomalous regions. As a practical demonstration, we deploy a robot manipulator to uncover clutter in the scene. We use the manipulator to push items to enable grasping and picking objects in densely packed environments. Our approach selects the optimal motion primitive which minimizes the perturbed amount of clutter, to highlight the ability of our detector to select meaningful anomalies in the environment. To the authors’ best knowledge, this is the first time a generalized hyperspectral and RGB-Depth anomaly detection framework has been developed and demonstrated on dynamically generated datasets.

The key contributions of this work are:

1. An algorithm to align and register multiple high-dimensional hyperspectral data cubes to RGB-Depth data.
2. A method for clustering of registered RGB-Depth-HSI fused data to create a coarse initial binary classification map.
3. A near-realtime training method to train a more complex autoencoder from the initial binary classification map without human intervention.
4. Identification and evaluation of heuristic-driven optimal motion primitives to uncover anomalies.

This paper is organized as follows. In Section 2 we discuss the current state of the art in hyperspectral and multi-modal anomaly detection with applications in robot perception. Our design for a multi-modal sensing array and robot workcell is presented in Section 3. Section 4 details the feature matching approach used to register a hyperspectral datacube to a 3D point cloud. Section 5 outlines our novel approach to use the fused data product to effectively identify anomalies in a cluttered environment and their associated confidence levels. Section 6 discusses the motion planning to generate and evaluate candidate paths to perturb piles in a cluttered environment. Section 7 documents the sample scenarios used to demonstrate our approach. Our results are detailed and discussed in Sections 8, 9, respectively. Finally we summarize the key experimental results and discuss future opportunities to extend our work in Section 10.

## 2 Prior work

Data-driven approaches to anomaly detection are not without their challenges. Increasing the number and types of sensors available to robots naturally increases the amount of data processed to make an inference at every time step. Correlating data acquired from different sensors, at different acquisition rates can also prove challenging. Moreover, each sensing modality has its own particular failure modes, and instances where failure rates or noise will be proportionally larger. For example, long wave infrared sensing is marked by poor texture resolution and highly uniform surfaces Zhang et al. (2021). Hyperspectral imaging is also dependent on adequate active illumination, and sufferers from slow acquisition times when compared to RGB cameras. While RGB cameras are rich in texture and resolution, their spectral resolution is fractional compared to HSI. In many robotics problems, sensors ultimately drive the capabilities, and limitations of the robot’s operations. Multi-resource anomaly detection requires the additional challenge of extracting meaningful features from each type of sensor, and developing frameworks to coalesce individual sensor features into a common operating picture.

### 2.1 Anomaly detection and tracking

Hyperspectral imaging originated as an airborne remote sensing technology with very coarse resolution. Therefore, great emphasis has been placed on per-pixel and sub-pixel anomaly detection. Reed and Yu (1990) first introduced a statistics based approach, by creating the popular Reed-Xiaoli (RX) algorithm, often used as a benchmark against novel approaches for anomaly detection. The RX algorithm assumes a multivariate Gaussian distribution of the background scene, performing adaptive constant false alarm rate detection derived from the generalized likelihood ratio test. However, in real world applications, the normal distribution of hyperspectral images is not probable as posited by Matteoli et al. (2010). To counteract this drawback, the RX algorithm is often deployed locally with a window such as in Taitano et al. (2010), considering a small portion of a larger image that is far more likely to exhibit Gaussianity. Zhao et al. (2015) consider this local focus in conjunction with a global view, extending previous work to create a real-time detector, that functions on small pixel anomalies. An alternative reconstruction is the collaborative-representation-based detector (CRD), developed by Li and Du (2015). This method exploits the phenomena that low-resolution anomaly pixels cannot be represented by proximal pixels, in direct contrast to non-anomalous pixels. The CRD method outperforms the RX algorithm Li and Du (2015); Su et al. (2022), but with exponentially longer runtime.

Deep learning has also gained traction as a form of anomaly detection in hyperspectral images. In Ma et al. (2018), the authors proposed an adaptive weight deep belief network that functions as an autoencoder (AE). By attempting encoding, and then reconstructing spectral signatures, the Euclidean distance between the two samples can be used to identify anomalies. Arisoy et al. (2021) also utilized an autoencoder network in conjunction with a generative adversarial network (GAN) to identify anomalous pixels from their reconstruction error. Xu et al. (2022) offers a comprehensive review of machine learning based approaches with added emphasis on deep methods. It is important to note that deep learning approaches are often difficult to introspect, creating adversity in troubleshooting and guaranteeing consistent behavior. Each of these prior studies rely on benchmarked, well-studied datasets and hence cannot cope with dynamic scenarios. Finally these algorithms make no claims of real-time performance, nor have they been demonstrated on datasets acquired at a close working distance where anomalies are much larger than a singular pixel.

Outside of pure hyperspectral imaging, other works have proposed the use of infrared wavelengths of light to provide enhanced target detection Zhang et al. (2021); Thananjeyan et al. (2022). The successful use of extended wavelength information to identify humans and materials in scenes demonstrates the utility of the technique and its applicability to different problem spaces Mao et al. (2022).

### 2.2 Hyperspectral-point cloud fusion

Hyperspectral data, providing material information, and point clouds, providing geometric information, yield a more complete picture of object function when considered together. Stemming from the 2013 IEEE Geoscience and Remote Sensing Society (GRSS) data fusion contest, HSI and point cloud fusion has become a richly researched field Debes et al. (2014). Rasti et al. (2017) utilized orthogonal total variation component analysis (OTVCA) to fuse the two datasets. Their works also incorporate extinction filters which can rapidly extract spatial features without threshold tuning. Khodadadzadeh et al. (2015) proposes another method based on morphological attribute profiles (APs) to build extended, reduced-order profiles of the spectral and surface at each pixel. Finally, Chen et al. (2019) demonstrated a unified system to simultaneously acquire a point cloud and hyperspectral image. The rapidly registered data was shown to be easily segmentable though the use of DBSCAN and manually-curated spatial features Ester et al. (1996).

### 2.3 Spectroscopy in robots

This research effort builds on our prior experience with spectroscopy for robots. In prior experiments, we designed and implemented a gripper system to acquire recursive estimation models of grasped items as an items was grasped by a parallel-plate gripper Hanson et al. (2022b). We have also applied spectroscopy to applications in soft robotics Hanson et al. (2022a) and mobile robotics Hanson et al. (2022c). In these studies we have found spectral signatures to be an extremely explainable metric for understanding abstract material types in the context of robotics.

Other groups have investigated the usage of spectroscopy to aid in household manipulation and tasks Erickson et al. (2019) and Erickson et al. 2020). These studies have focused on the usage of a point-based spectrometer to acquire a single spectral signature to classify the base material of a scanned item prior to manipulation. However such an approach is not optimal as only a small surface area of each item can be scanned, making coverage times for whole spatial scenes intractable in time and power consumption.

Hyperspectral imaging has found widespread acceptance as a tool in machine vision for classification of fruits, waste products, and in many other organic and inorganic items Barnabé et al. (2015); Ku et al. (2021); Kwak et al. (2021). These approaches demonstrate the diversity of applications the technology is relevant to, but lack generality to multiple robotic problems. These past works rely heavily on pre-trained, supervised classification models to correctly identify known foreign contaminants or other undesirable object states such as disease, wilt, discoloration. The high accuracy achieved by Vali et al. (2021) shows encouraging results for most non-linear classifiers to yield high accuracy, but still with the burdensome requirement of curated labels.

## 3 System architecture

**FIGURE 1:**
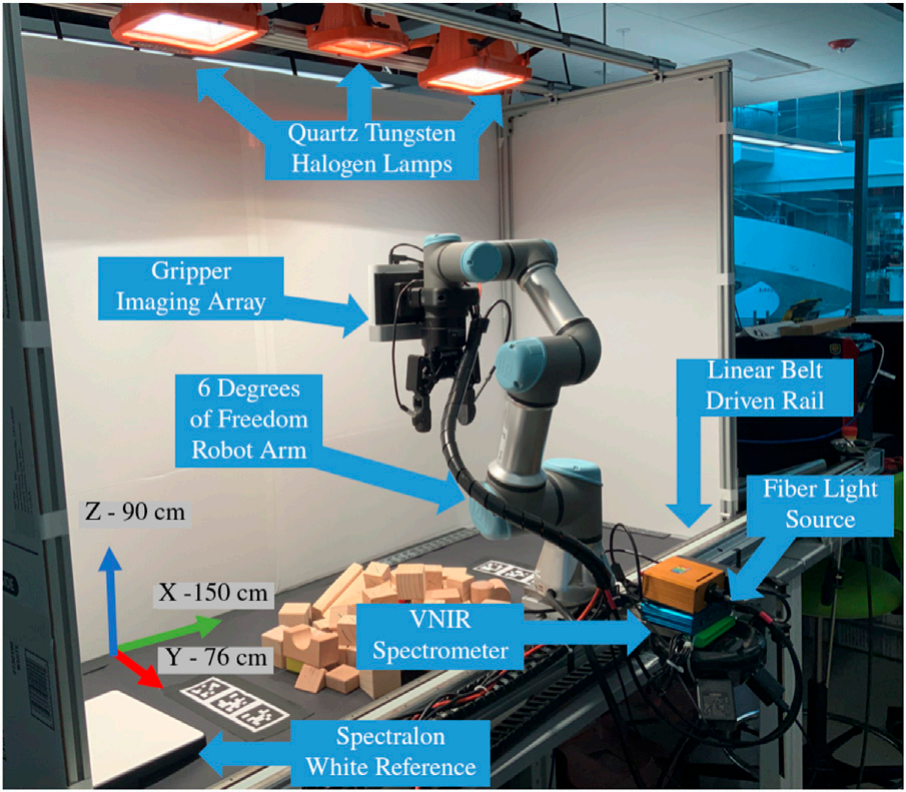
Test bed architecture used to acquire and register multiple Visible Near-Infrared (VNIR) hyperspectral scans for analysis. Note the axes orientations and workspace size.

As a precursor to the algorithm, and technical development, it is necessary to outline the robot testbed and its capabilities. For fuller details on the system design, our prior work offers detailed instructions to replicate the robot workcell. Hanson et al. (2022). The full system diagram is annotated in Figure 1.

### 3.1 Robot manipulator

At the core of our setup was a 6 Degree of Freedom (DoF) robot manipulator (Universal Robotics). When operating the arm, we represented the end effector pose with a vector $x_{n}\in R^{6}$ with the first three elements allocated to the 3D position, $x_{p}^{n}\in R^{3}$ , and the latter to a quaternion representation of rotation $x_{n}^{q}\in R^{4}$ . The base of the robot was established at the origin of the global coordinate frame. The end effector pose, relative to this base frame was determined through a forward kinematic chain. This kinematic modeling was used to both position the arm for capturing multiple images of the workcell scene (Section 4), and planning uncovering motions (Section 7). The robot was controlled through the Robot Operating System (ROS) software interface *via* a Linux PC Quigley et al. (2009). The end effector contained a modified finger parallel plate gripper (RobotIQ) with optically clear finger pads, which will be utilized in future research.

### 3.2 Perception

**FIGURE 2:**
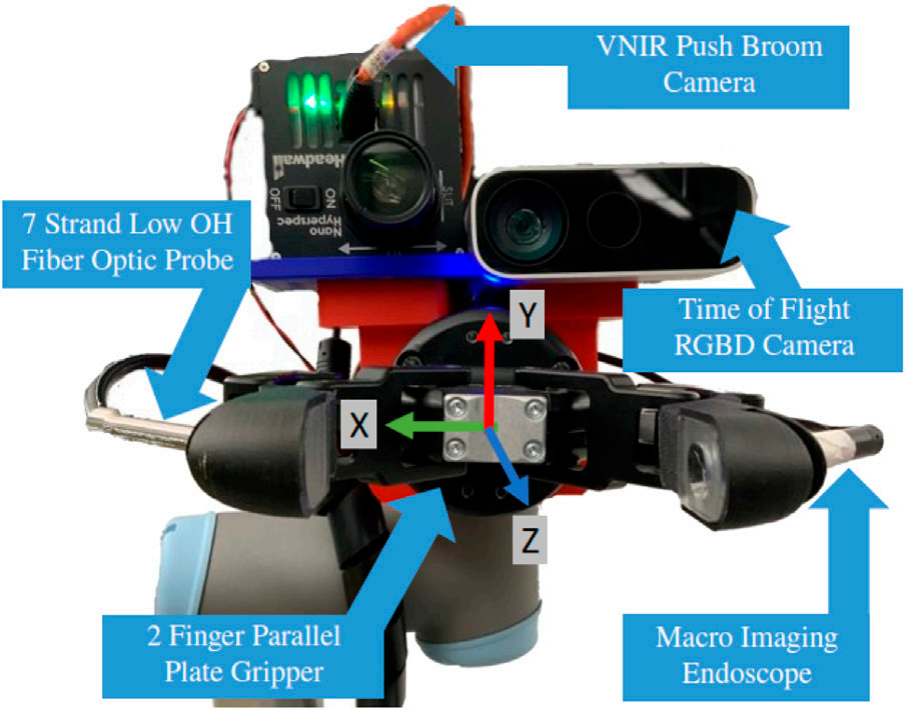
Sensor array fitted to end effector of manipulator arm, with sensory systems for both scene and in-hand object sensing. *NB*: *Z*-axis is perpendicular, coming out of the page.

On the last link of the robot arm, we mounted two sensors to capture high resolution spatial and spectral information. The first sensor, a Time of Flight (ToF) depth camera (Microsoft). At the nominal operating height of 0.7 m above the workcell surface, the ToF camera was estimated to have a systematic spatial error ≤1 mm, making it perfect for perceiving the presence of small objects in scene Kurillo et al. (2022). The camera outputs registered RGB and depth images; the latter of which can easily be projected into 3D space relative to the robot base with the TF package Foote (2013). The sensor layout is presented in Figure 2.We co-aligned a VNIR pushbroom hyperspectral camera (Headwall Photonics) with the lens of the ToF camera. Unlike typical RGB image formation which integrates a spatial scene over a single timestep, pushbroom cameras need motion to generate a 2D spatial image. This was achieved by moving the camera and stitching subsequent rows into a singular spatial scene. The exposure time was set to 60 ms per line image and the system moves at 0.01 m/s.

### 3.3 Linear rail

The main robot assembly was mounted to a linear rail (LOPRO) driven by a high precision stepper motor. The rail provided a steady means to move the robot arm at a fixed rate along a linear path in the *y*-axis. This motion is critical to the successful operation of the hyperspectral camera, as the usable rail length (1.0 m) exceeded the Cartesian path capabilities of the arm ( ≈ 20 cm), while maintaining the end effector in a constant orientation. For usage with the hyperspectral camera, the rail was operated at a velocity of 1 cm/s. This optimal velocity value was empirically determined by measuring pixel size distortion in the pushbroom image formation process and accounting for the number of detected photons per integration period.

### 3.4 Illumination

Good scene illumination is integral to hyperspectral imaging. Normal Light Emitting Diodes nominally cover ≈20nm Full Width Half Maximum (FWHM) Weik (2001) wavelength range, making them poor candidates for full scene illumination across the VNIR spectrum. We circumvent a complicated LED lighting display by using overhead mounted Quartz Tungsten Halogen (QTH) work lights. QTH bulbs provide uniform intensity illumination between 350–2,500 nm and are regularly used in spectroscopy for material interrogation. The lamps are rated at 250 Watts and output of 4,000 lumens each. Although the lights do generate a significant amount of heat, that thermal energy is primarily convected upward resulting in only minimal heating of the surface. No heat related compensation is necessary in processing the raw hyperspectral datacube.

### 3.5 Computation

A core motivation for this research is anomaly detection algorithms congruent with reasonable computer requirements. All algorithms were developed in Ubuntu 20 on a desktop PC with 32 gigabytes (GB) Random Access Memory (RAM) and 8 processing cores. For training the autoencoder network, we used an NVIDIA GeForce GTX 1070 GPU to expedite training and offload computation from the CPU.

## 4 Data registration

A depth image as well as RGB-image of the entire workspace can be obtained from the ToF infrared camera instantaneously from the home position of the arm, with the robot base located at {0,0,0} in the global X,Y,Z coordinate frame. The sensor’s field of view allows for the imaging of the entire scene in a single image. In contrast to the ToF camera, the hyperspectral push-broom sensor has a horizontal field of view along the *x*-axis, requiring both linear motions and repositioning of the arm to characterize the entire workspace. For our calibration procedures, we constructed a working environment out of piles of unpainted wooden blocks of varying size and shape, constituting the “pure” material. We mixed in smaller colored blocks, made of both plastic and wood as anomalies.

### 4.1 Data collection

To collect a scan, the system was initialized with three pre-planned end effector positions covering the top, middle, and bottom sections of the robot workcell. The chosen manipulator’s small workspace constrained the coverage to three parallel scans offset by 10 cm along the *y*-axis, but the methods presented in this section are generalizable to any number of scans. In each position the hyperspectral camera and ToF camera were aligned so their lenses are perpendicular to the surface plane. Holding this assumption simplifies the data association task by forcing image registration to be dominated by affine transformations. The inverse kinematic planning to each of these position was solved using the Levenberg–Marquardt algorithm Levenberg (1944). With the joint angles from the solution, the robot was commanded through ROS to move to set overhead scan positions.

In each scan configuration, the linear actuator translated the robot arm and imaging array over the cell contents at a speed of 1 cm/s. A preallocated memory buffer was used to read incoming hyperspectral linescans and stack them into a singular image. The speed at this stage was precisely controlled to minimize stretching or compression induced by inconsistent scan speeds. Each image in its raw format is approximately 2.8 gigabytes (GB). After the scan was completed, the image was written to disk to avoid excess storage of data in the system RAM.

### 4.2 Image preprocessing

We first preprocess the raw hyperspectral sensor data. Association requires the generation of image features and descriptors. Since preprocessing is particularly challenging due to limited spatial resolution of the hyperspectral camera relative to the RGB camera, we first normalize the hyperspectral image according to the equation presented in Eq. 1. S_raw_ is a row from the hyperspectral camera (640 × 273 pixels). D_HSI_ is the dark sensor reading averaged for each photodetector pixel over 100 lines of acquisition. Normalization allows us to account for sensor noise caused by temperature and system intrinsics. L_HSI_ is an averaged sensor reading over a sheet of Spectralon white reference material, which reflects 99% of incident light in the VNIR range Bruegge et al. (1993). The Spectralon reference provides and upper limit for what ≈100% reflectance looks like for each pixel subject to the current environmental lighting conditions.

$${\text{S}}_{\text{cal HSI}}=\frac{{\text{S}}_{\text{raw}}-\min\left({\text{D}}_{\text{HSI}}\right)}{\max\left({\text{L}}_{\text{HSI}}\right)-\min\left({\text{D}}_{\text{HSI}}\right)}$$ (1)

Next, we select bands constituting reflectance at the peaks of the visible Red, Blue, and Green reflectance as 707.48 nm, 477.27 nm, and 537.04 nm, respectively. Together these images yield an RGB representation of the data cube which is used in feature extraction. This image is then converted to grayscale by averaging the channel intensities together.

The hyperspectral sensor prioritizes spectral over spatial resolution, resulting in degraded borders and fuzzy features which would ordinarily frustrate detection of reliable image features. We first remove image noise by adopting the formulation provided by Buades et al. (2011). We assume the measured value of each pixel is represented by $p=p_{0}+N\left(0,\sigma \right)$ where *p*_0_ is the true intensity and is subject to zero-mean Gaussian noise. This step importantly removes image speckling which might otherwise create false features in the spectral signatures.

Next we sharpen the image using a convolved window function, following Eq. 2.

$${\text{Img}}_{\text{sharp}}={\text{Img}}_{\text{original}}+\left({\text{Img}}_{\text{original}}-G⊛{\text{Img}}_{\text{original}}\right)*\kappa$$ (2)

**FIGURE 3:**
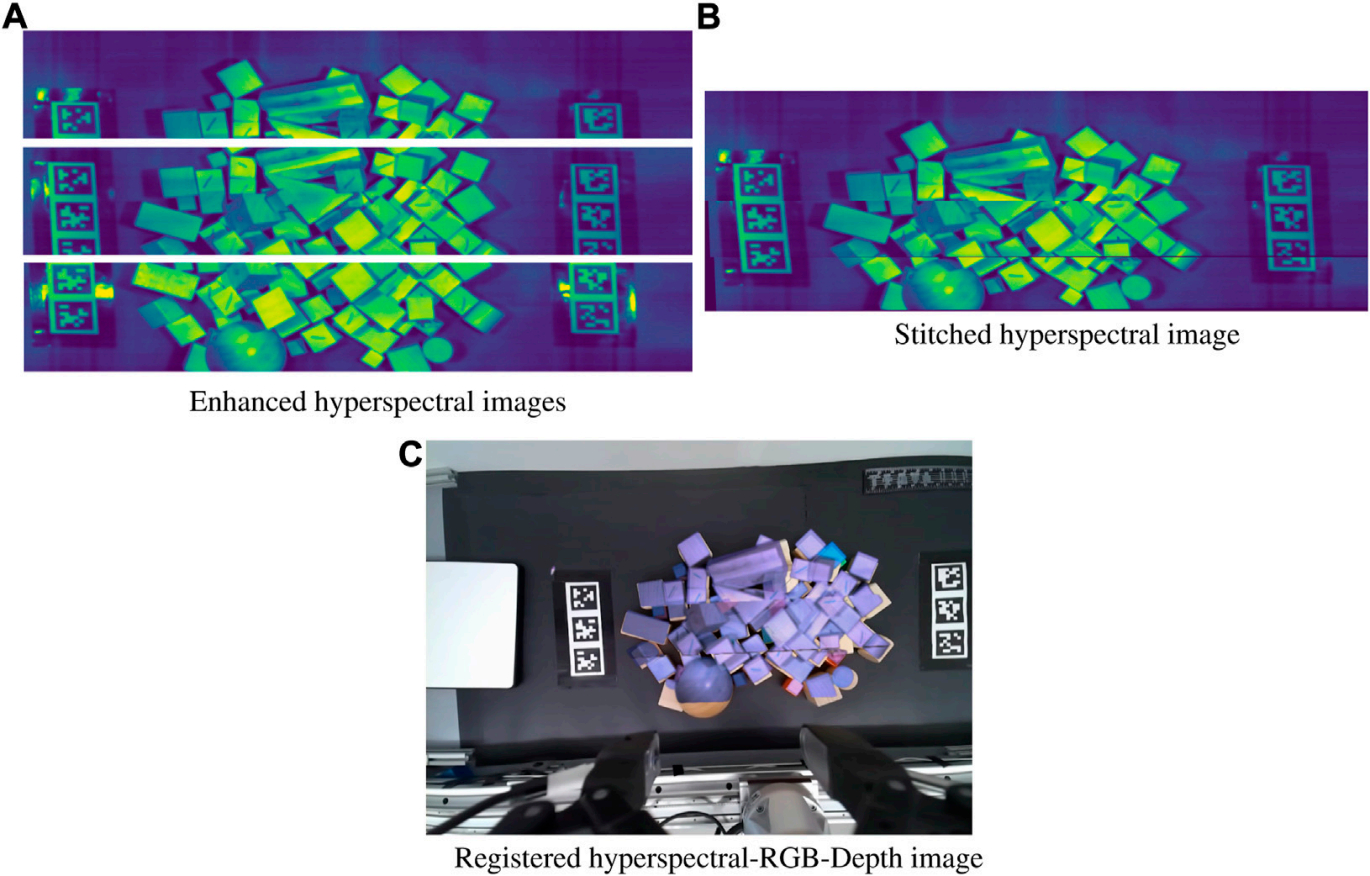
Image registration with **(A)** Noise filtering, sharpening, and CLAHE applied to each respective image **(B)** Enhanced images stitched together from shared features and homography **(C)** Stitched hyperspectral image warped to directly correspond to the RGB image from shared features, and overlaid for visualization.

*κ* represents the proportional amount of the blurred image difference to remove. *G*_*i*_ is a Gaussian filter of set kernel size and width. Finally, we apply Contrast Limited Adaptive Histogram Equalization (CLAHE) which ensures localized contrast areas Zuiderveld (1994). This step is not strictly necessary when the image has multiple different objects present but is aids when the scanned surface is relatively homogeneous as CLAHE can emphasize local features that were previously obscured by global image intensity. The individual steps of the image cleaning process are shown in Figure 3.

### 4.3 Feature detection and matching

**FIGURE 4:**
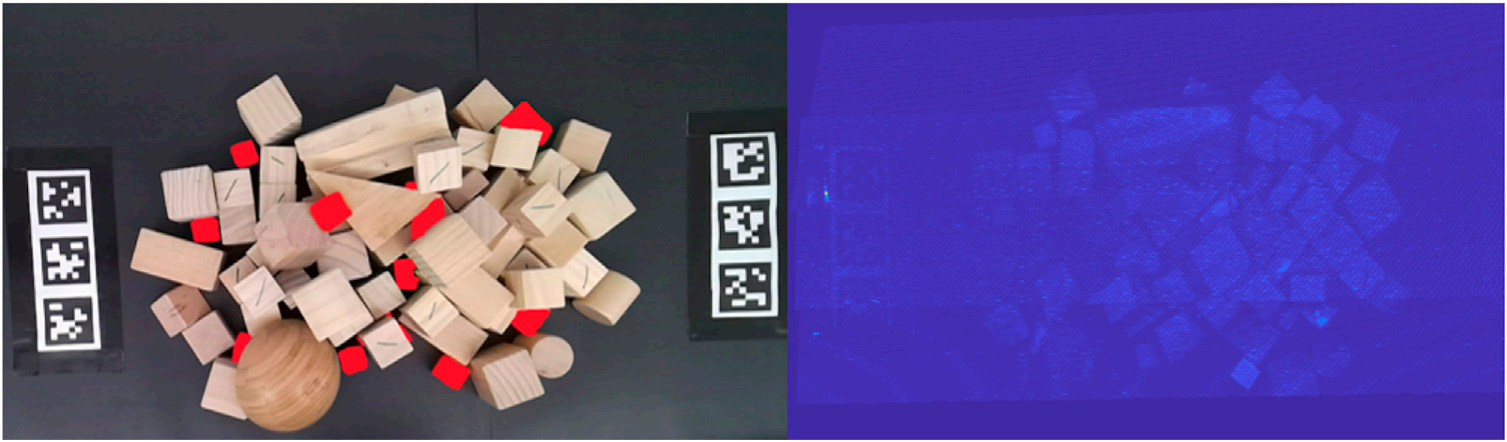
The blocks labeled with red highlights showing ground truth regions that should be detected as anomalous and distinct from the background. When using RX as the statistical anomaly detector, the colorized outline scores are shown on the right. The RX outlier detector fails to distinguish anomalous regions from the pure wooden blocks.

Our process entails the mosaicing of individual image scans to each other, before the registration to the full scene RGB image. Distinct features in all images are determined through using SIFT Lowe (2004). We also considered the ORB feature detector Rublee et al. (2011), but found the distribution of features across the image was confused by the poor image resolution. The Fast Library for Approximate Nearest Neighbors (FLANN) Muja and Lowe (2014) is used to identify and score candidate matches between images. Using Lowe’s ratio test, we can eliminate ambiguous features matches by considering the L2 norm between both features as source and destination matches Lowe (2004). Our usage of SIFT generates 1,000 candidate keypoints in each image, although the number of matches depends on the scene contents. The matched points are assumed to be co-planar, meaning we can warp the two images together with the formula given in Eq. 3. *H* is a 3 × 3 homography matrix, *c* is a scale factor, and the two matrices represent pixel coordinates Figure 4.

$$\left[\begin{array}{c} \hat{x_{i}}\\ \hat{y_{i}}\\ 1 \end{array}\right]c=H_{1\to 2}\left[\begin{array}{c} x_{i}\\ y_{i}\\ 1 \end{array}\right]$$ (3)

Random Sampling Consensus (RANSAC) Fischler and Bolles (1981) assists in the estimation of the homography matrix from matched points {*x*_*i*_, *y*_*i*_}, regardless of potential image noise or feature poor feature matches. This step is essential as the image formation process is subject to system noise, causing the length to vary from one scan to the next, typically by ± 10 pixels.

We start the association with the center image and work outward iteratively registering images to the center mosaic. As we combine images, we calculate an additional translation needed to keep the warped image fully in the bounds of the new image canvas: *H*′ = *H* ⋅ *t*, storing the perspective transform of the cumulative hyperspectral image with each added hyperspectral image. The same method is repeated to register the hyperspectral mosaic to the RGB method. We found Gaussian blurring the RGB image yields better matches with the lower-resolution hyperspectral scene. In images with weak features, the fiducial markers Wang and Olson (2016) on the borders of the scene greatly aid in the successful registration of the image. The homographies determined from comparing the RGB representation datacubes among themselves and the RGB Kinect image are applied to each channel of the hyperspectral datacube. This results in warped and translated datacubes, which are added to the same final canvas, resulting in the a single RGB-D-Hypersectral data representation used in the rest of this work.

This image registration step holds an assumption that the scene held with be largely planar. This is reasonable given that the imaging array is translated linearly above the object surface at a fixed height. The selected objects for use in this study do not exceed a height greater than 10 cm above the surface plane. Although we do observed some small distortions around taller objects, the homography can be quickly calculated and results in a 2D representation of the scene, aligned with the Kinetc-generated depth and RGB images. Treating these points as 3D would then require reprojection into a 2D frame for correlation with the Kinetc image, thus further complicating the reconstruction. The final perspective transform results in some spatial data reduction as the hyperspectral scan length, typically 4,000 pixels, exceeds that of the RGB image. The final data product includes three equally sized images: the hyperspectral mosaic, the scene RGB image, and the registered depth image. From the depth image, we are able to generate a point cloud for object manipulation.

## 5 Anomaly detector

In order to achieve good unsupervised model results, we make assumptions regarding the composition of the workspace. We assume that the two dominant material abundances will be the background and the pure objects. We also assume the presence of anomalies will never constitute more than one-third of the total visible item surface area in the workspace. The novelty of our approach comes in a two stage detector leveraging both classical clustering techniques, and then an autoencoder network trained in real-time to rapidly identify anomalies.

### 5.1 Clustered anomaly detection

Initially, we attempted to use a global RX detector for anomaly detection. This statistic based approaches considers spectral dissimilarity of pixels relative to the background of the scene. Given a set of pixels, with their full spectrum wavelength values, the RX score for each is calculated following Eq. 4.

$$\delta_{\text{RX}}\left(x\right)={\left(x-\mu \right)}^{⊺}{\left(\frac{\Sigma \left(x_{i}-\mu \right)}{N}\right)}^{-1}\left(x-\mu \right)$$ (4)

*x* represents the current pixel vector; *μ* is the average spectral signature; *N* is the total number of pixels in the image, however, large clusters of anomalous pixels confuse the classical RX global anomaly detector. Following the results of Su et al. (2022), we note that local patch-based anomaly detectors, such as CRD and LRX 2 will only further exacerbate runtime problems. In our RX results, we observed very poor detection of the anomalies due to their multiple pixel presence Figure 4.

Therefore, our reformulation of the base detector problem is as follows. We begin by assuming the environment consists of four primary classes: {no-signal, background, base material, anomalies}. No-signal constitutes all regions where there is no co-registered hyperspectral data; background indicates regions where there is hyperspectral data but the dominant signal is the black table mat; base material is the “pure” material to which all anomalies should be considered; anomalies are all other regions not fitting the three prior descriptors. This presumed decomposition follows the following ranking of cardinality: |anomalies| ≤ |base material| ≤ |background| ≤ |no-signal|.

**FIGURE 5:**
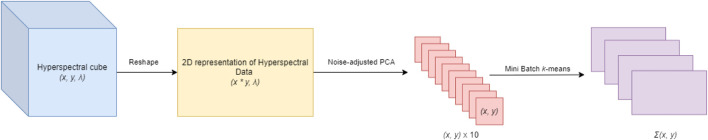
The data segmentation pipeline showing the flow of data during the initial segmentation.

**FIGURE 6:**
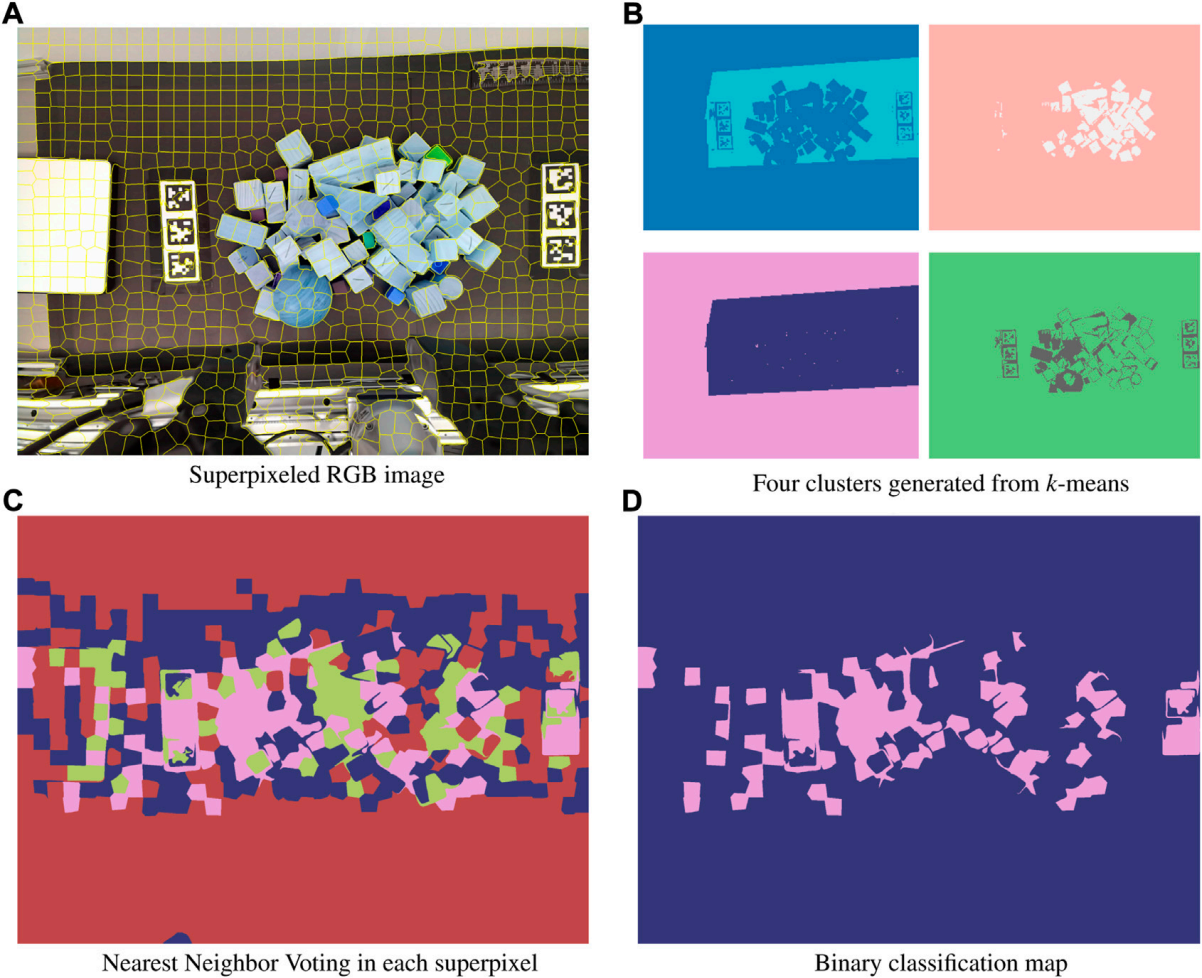
The process of cautiously creating definitively pure data to train the AE is as follows: First, superpixeling is used to split the RGB image into characteristic regions **(A)**. k-means segmentation in abundance classes from the hyperspectral datacube is applied to segment the associated RGB-HSI data into four clusters **(B)**. The dominant cluster within each superpixel, biased to favor potentially anomalous regions, is identified **(C)**. This information is then used to create a binary classification map, with the two labels being pure regions and potentially anomalous regions, the latter of which is excluded in the autoencoder training process **(D)**.

Simple heuristics here can guide the selection of candidate regions for these classes. Visual reference for the following procedure is given in Figure 5. First, we decompose the *x*, *y*, *λ* datacube into a 2D representation x*y, *λ*. Next, we consider datacube’s wavelength channel is often redundant and not fully necessary to explain the total variance in the image. Minimum Noise Fraction (MNF), also known as noise-adjusted Principal Components Analysis (PCA) is used to perform a change of basis and reduce the wavelength channel dimensionality Lee et al. (1990). We heuristically selected 10 components as the number of components to retain, as this exceeds the number of expected classes by a factor of 2.5. Here we apply mini batch *k*-means clustering to segment the scene into four regions. Prior dimensionality reduction provides the added benefit of faster cluster convergence, as the number of features needing comparison decreases by an order of magnitude through MNF. The number of pixels belonging to each class is counted, and the size-ordered regions are given the initial labels following the previously discussed ranking. Visual inspection of Figure 6B reveals adequate clustering the background and no-signal, and base material classes, but generally poor performance within the anomaly class. As long as the base material is still accurately dominated by the true base material, erroneously assigned pixels in anomalous regions will not significantly impact the final classification.

### 5.2 Superpixel region creation and evaluation

As a result of *k*-means considering pixels in isolation, pixels lose out on surrounding neighborhood information. Isolated pixels may be erroneously assigned to the wrong initial class, thus skewing the correct assignment of the data. To regain spatial information and avoid erroneous pixels, the overhead RGB image of the workspace obtained from the Kinect ToF camera is decomposed into multi-pixel regions called superpixels. Superpixeling creates indicative pixel conglomerates to analyze for anomalies calculated through clustering which is simpler than evaluating each pixel for anomalies on an individual basis. We empirically determined 1,000 superpixels was a sufficient number given the size of the composite image. Superpixels under this strategy generally consist of 3,800 pixels, with an average spatial dimension of 3 cm × 3 cm. Our implementation uses Simple Linear Iterative Clustering (SLIC) Achanta et al. (2010), which converts images to the CIELAB color space before iteratively updating cluster centers. The distance function for determining cluster centers is enumerated as:

$$\begin{array}{c} d_{c}=\sqrt{{\left(l_{j}-l_{i}\right)}^{2}+{\left(a_{j}-a_{i}\right)}^{2}+{\left(b_{j}-b_{i}\right)}^{2}},\\ d_{s}=\sqrt{{\left(x_{j}-x_{i}\right)}^{2}+{\left(y_{j}-y_{i}\right)}^{2}},\\ D_{\text{total}}=\sqrt{d_{c}^{2}+{\left(\frac{d_{s}}{S}\right)}^{2}m^{2}} \end{array}$$

*l*, *a*, *b*, are values from the LAB color space conversion; *x*, and *y* are pixel coordinates, *S* is the average desired size of superpixels, in number of pixels, and *m* is a scale factor for the relative importance of color and spatial distance factors. This approach comes with the consequential assumption that superpixels will have relatively homogeneous visible color values. This assumption minimizes the time needed to generate regions of reasonable purity, using a subset of the hyperspectral camera range. We avoid overvaluing the contributions of color by favoring *d*_*s*_ by a factor of 10. Implementing a similar SLIC algorithm on the HSI data cube would include a distance function over 91 times more data per image pixel. Figure 6A shows the distribution of superpixels in a sample image scene.

For each superpixel, we count the constituent class members, understanding the superpixels might contain several regions. Using majority voting biased towards anomalies we then reclassify each pixel as “pure” or “impure” according to the following binary classification rule:

$$\hat{x}=\text{argmax}\left(0.80*\left(\left|\text{no}-\text{signal}|+|\text{background}|+|\text{base material}\right|\right),|\text{anomalies}|\right)$$

where $\hat{x}$ is the predicted class. The value 0.80 is an empirically determined constant that diminishes the weighting of non-anomalous pixels in the superpixel. This calculation is enabled through the aforementioned one-to-one mapping of stitched hyperspectral images to the RGB image using homography, where each pixel’s location is the same across hyperspectral and RGB images. Figures 6C,D show the majority voting and application of the binary classification rule. Weighted majority voting prevents the accidental inclusion of anomalies in the training of the autoencoder, detailed in the subsequent section.

### 5.3 Autoencoder

The *k*-means method constitutes a weak learner since it leverages assumptions and heuristics to create general separability in the scene data. The method is also designed to avoid overfitting description of the data. Namely, the classification rule for pure vs. impure is intentionally biased to avoid regions with potential impurity. The next stage in our detector pipeline uses an unsupervised autoencoder to provide per pixel scoring of anomalies in the environment.

The autoencoder works on a simple principle: by learning a low-dimension representation of the data (encoding), and then reconstructing the signal (decoding), the error in reconstruction can be used to assess the presence of anomalies. Because the encoding layers in the network are fully-connected, the latent space representation is a non-linear combination of the individual features of the input spectral signature. This contrasts with other dimensionality reduction techniques like Principal Components Analysis (PCA) or Non-negative Matrix Factorization (NMF) which provide a strictly linear combination of the input features.Formally we define the structure for our network:

$$\begin{array}{l} \phi :X\to S\\ \psi :S\to X\\ \phi ,\psi =\underset{\phi ,\psi }{\mathrm{argmin}}\|X-\left(\psi \circ \phi \right)X{\|}^{2} \end{array}$$

where X is the input signal such that $x\in R^{273}$ , and S is the latent space given that dim(S)<dim(X) . *ϕ* is the encoder network, and *ψ* is the decoder network. When trained, we use a mean-squared loss function to construct the network weights of the encoder and decoder.

**FIGURE 7:**
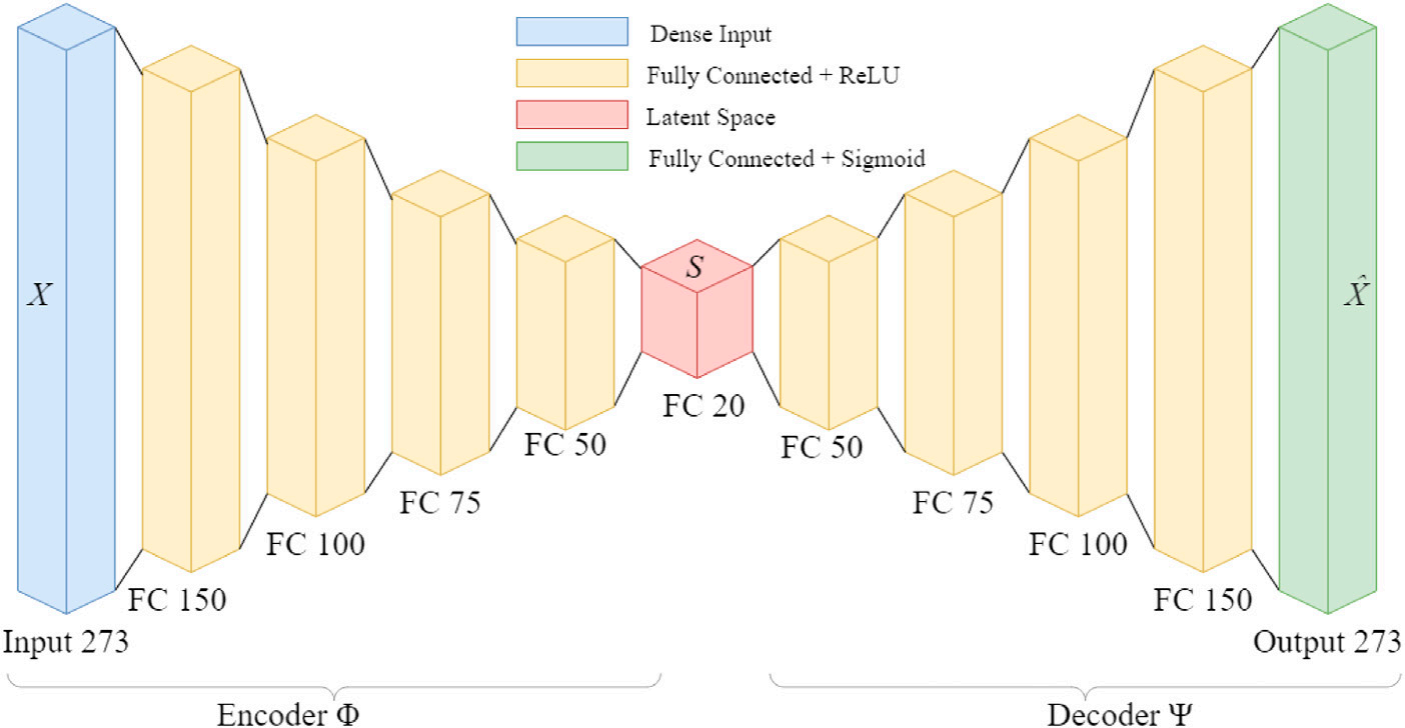
Network architecture for fast hyperspectral anomaly detection. The input data is reduced by an order of magnitude in the latent space, before reconstruction into the original dimension.

**FIGURE 8:**
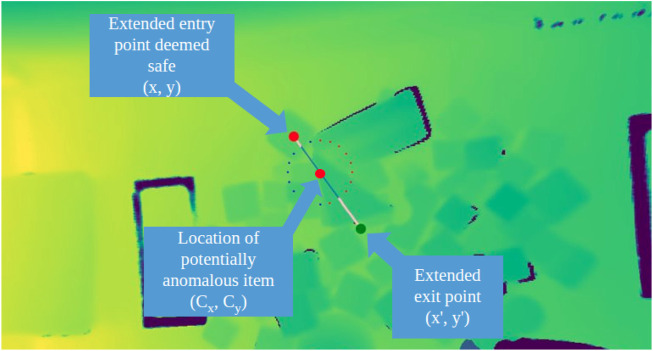
Generation of optimized motion primitive to perturb object clutter. The circle shows the radially generated candidate motion plans. In the situation, the entry and exit points of the plan are modified to prevent collisions with the gripper as it descends into place.

The pure regions of high certainty from the hyperspectral image are used as training input for the autoencoder. The encoder network decreases the data size from a 273 unit vector to a 20 unit vector. The network was implemented in Python using TensorFlow. The network consisted of fully-connected layers followed by a Rectified Linear Unit (ReLU) Nair and Hinton (2010). The network architecture is illustrated in Figure 7 The model was trained for 20 epochs and utilized early stopping to exit model training in situations where the model failed to improve after five consecutive epochs. Using the compute setup detailed in Section 3.5 each epoch takes an average of 3 min to train, and 10 s to evaluate on the full datacube. After training the model weights are saved for later reuse in model evaluation on similar datasets. Although this network is shallow, the reduction in weights greatly expedites the training time and prevents the network from simply memorizing the existing data. This network is trained immediately following the initial binary segmentation, without tuning by a human operator. This real-time training results in a faster generation of a deployable anomaly detector compared to one that requires copious hyperparameter tuning and expert systems input Figure 8.

Once generated, anomalous pixels are scored by considering the Mean Absolute Error of the reconstruction loss:

$$L\left(x,\hat{x}\right)=\frac{\sum_{i=1}^{n}|x_{i}-\hat{x_{i}}|}{n}$$

After training, the autoencoder is optimized to perfectly reconstruct spectra of the majority class, which dominated the distribution of training data. Anomalies, which are not particularly abundant, are reconstructed with a high degree of error.

## 6 Motion primitives

With the results from the anomaly detection pipeline, the system shifts to focus around motion primitives to disturb the areas where anomalies are located. Grasp selection is another challenging problem in cluttered environment. Selecting the optimal attachment point requires understanding of the 3D structure and segmentation of items from one another. Moreover, grasping with a rigid gripper applies direct force, squeezing items as they are manipulated; potentially damaging deformable items. For these reasons, in this research we constrain our actions to pushing, instead of retrieval actions. Our approach is comparatively gentler and shows good generality to different environments. In executing a push, the two-finger gripper is closed to create a single contact point, providing more precisely controllable impact points than are capable with the open-jaw gripper. Given an anomalous region, we calculate its centroid under the assumption the polygon region is closed and non-intersecting. In this formulation, *x* and *y* represent the vertices of the polygon superpixel.

$$\begin{array}{l} C_{x}=\frac{1}{6A}\overset{n-1}{\underset{i=0}{\sum }}\left(x_{i}+x_{i+1}\right)\left(x_{i}y_{i+1}-x_{i+1}y_{i}\right),\\ C_{y}=\frac{1}{6A}\overset{n-1}{\underset{i=0}{\sum }}\left(y_{i}+y_{i+1}\right)\left(x_{i}y_{i+1}-x_{i+1}y_{i}\right),\\ A=\frac{1}{2}\overset{n-1}{\underset{i=0}{\sum }}\left(x_{i}y_{i+1}-x_{i+1}y_{i}\right) \end{array}$$

From the center point, cartesian paths are generated radially from the center at intervals of $\frac{\pi }{12}$ radians between paths. These plans are candidates which are heuristically scored by the volume of the cells they will cross. Intuitively, this means the robot will select the path which disturbs the smallest volume of clutter, and will increase the visible surface are of the occluded objects. The path selection is optimized by minimizing the intersected volume. The volume of the intersected superpixel is numerically estimated using a double trapezoidal integration of the *z*-axis values.

x,x′,y,y′=argmin∑in∬Vdidx dy (5)

*x*, *y* indicates the start of the motion path, and *x*′, *y*′ indicates the end of the path. The summation is conducted over all intersecting cell values, and *d* is the depth values for the *ith* superpixel. If the start position places the gripper above the clutter, then it is further improved by extending the point radially along the same line, until a point with more open space is found. Minimizing the impacted object volume enforces a constraint that motions should directly target the anomaly and its vicinity, rather than perturbing a large path crossing the entirety of the object pile Figure 8.

Upon selecting the optimized motion plan, the pixel coordinates are converted to a world coordinate frame using a pin-hole camera model. The arm first moves to *x*, *y*, *z* + offset where the offset is a parameter, set to 10 cm for the following experiments. The arm then descends to *x*, *y*, *z* and begins to move in a cartesian path to *x*′, *y*′, *z*′. The arm then returns to the home position, and the cycle repeats itself until the anomalous regions have been depleted.

## 7 Experiments

To validate our modeling approach, we constructed several challenging scenarios for the robot to operate in. Namely each scenario consisted of one predominant object, with variations in size, shape and coloration. Anomalies consisting of reasonably considered debris and detritus were added to the scene. Similarly, these objects also possessed considerable variance in their shape, color, and optical transmission. The specific scenarios consisted of the following setups:

• Plain natural wood blocks with painted, color blocks and plastic building block pieces both partially occluded.
• Real green vegetation mixed with faux plastic leaves.
• Lemons contaminated with small fragments of plastic from a shopping bag.
• Real lemons mixed with visually identical fake lemons, and yellow-green limes.

These scenarios serve as demonstrations of the algorithms ability to learn anomalies with limited examples. Specifically the mixing of visually indistinguishable real and faux items challenge traditional RGB-based methods.

**FIGURE 9:**
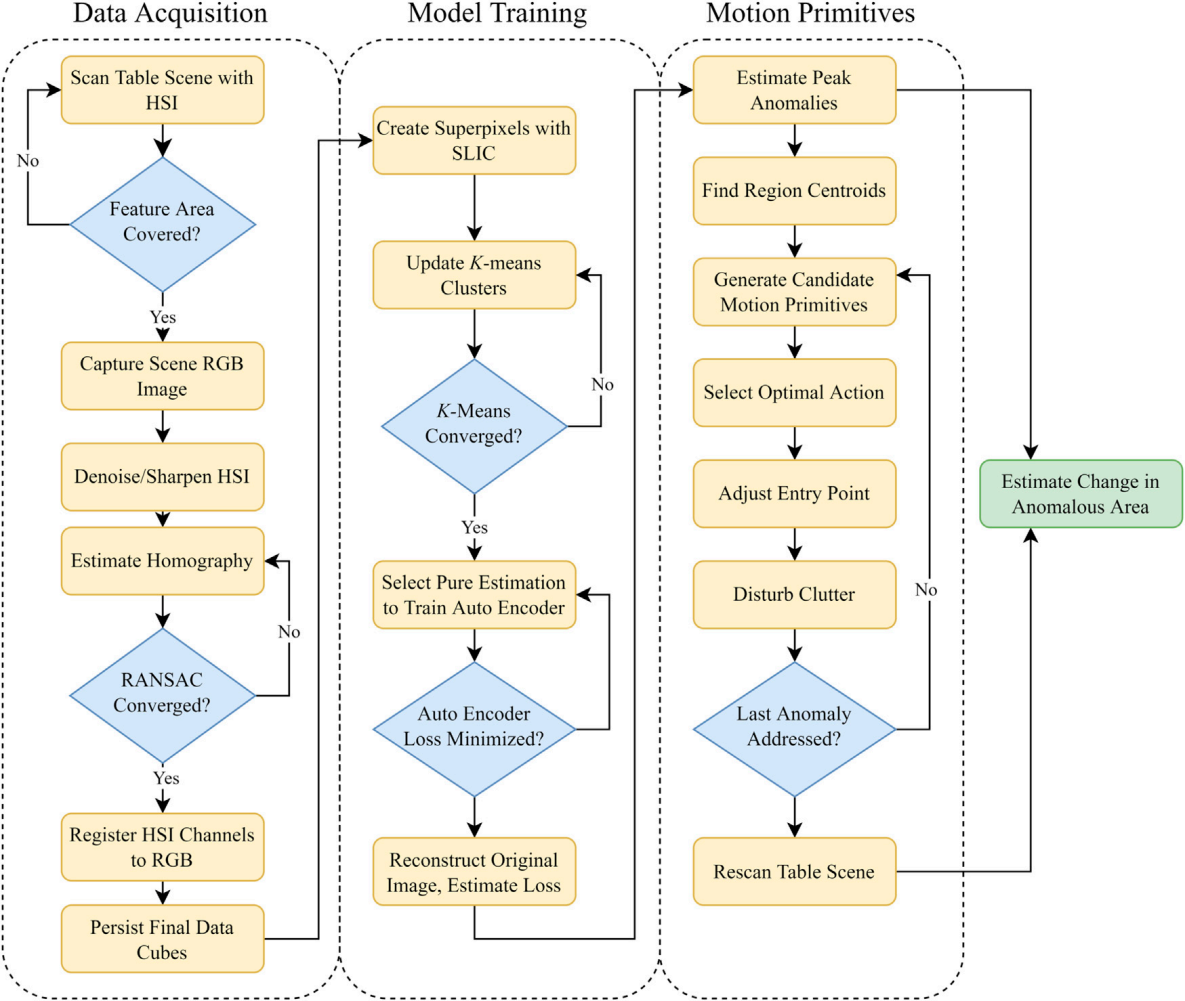
Three stage process for automated anomaly detection and uncovering. The data workflow is separated into three categories: data registration, anomaly modeling, and motion primitives.

For each setup, the items were pseudo-randomly distributed on the table, with some manual reordering undertaken to increase the environment complexity. The items were then scanned following the previously delineated procedure and a single normalized hyperspectral datacube was generated. After fitting and applying the unsupervised two-stage anomaly detector to the environment, candidate regions of interest were generated. The three most likely anomalies were selected and minimally disruptive motion primitives to each point were planned. After executing the three motions, the scan process was repeated to generate an after-action hyperspectral datacube. We intentionally limited the number of allowed pushing motions to three to minimize over-perturbing the items. The end-to-end workflow to detect anomalies with the HSI camera is show in detail in Figure 9.

## 8 Results

In performing our analysis of the effectiveness of the pipeline, we decompose the system into two parts:

• Effectiveness of anomaly detector under partial occlusions
• Effectiveness of the motion primitives in exposing occluded clutter

### 8.1 Anomaly detector

The two-stage anomaly detector was evaluated as followed. After running through the workflow presented in Figure 9, we saved the results collected from the autoencoder, the pixel-wise reconstruction loss, and used prior knowledge of the environment to create polygon labels for the anomalous regions in both the pre and post disturbance scene. Given a pixel *s*, in the data cube *d* the anomaly score is calculated by

$$\text{norm}\left(s\right)=\frac{s-\min\left(d\right)}{\max\left(d\right)-\min\left(d\right)}$$

This formula normalizes the anomaly scores in a range between 0 and 1. The data. Once the normalized value is obtained, the score used in determining final probability of anomaly is given by:

$$\text{score}\left(s\right)=\frac{\text{norm}\left(s\right)-\mu }{\sigma }$$

Here *μ* represents the mean value of all the normalized data, and *σ* is the standard deviations of the normalized values. Essentially, here we treat the likelihood of a pixel being anomalous as the number of standard deviations a pixel is from the mean. These values are used to observe the overall performance of the anomaly detection workflow.

As a metric for quantifying the performance of the system we consider the True Positives (TP), True Negatives (TN), False Positives (FP), and False Negatives (FN). We define TP as considered anomalous material that is correctly identified, TN as “pure” regions that are correctly identified, FP as pure regions that are incorrectly classified as anomalous, and FN as regions that are anomalous that are identified as pure. These individual categorizations are used to create metrics for the overall performance of the system. Specifically, we calculate the following metrics for each scene:

$$\begin{array}{l} \text{Precision}=\frac{TP}{TP+FP}\\ \text{Recall}=\frac{TP}{TP+FN} \end{array}$$

**FIGURE 10:**
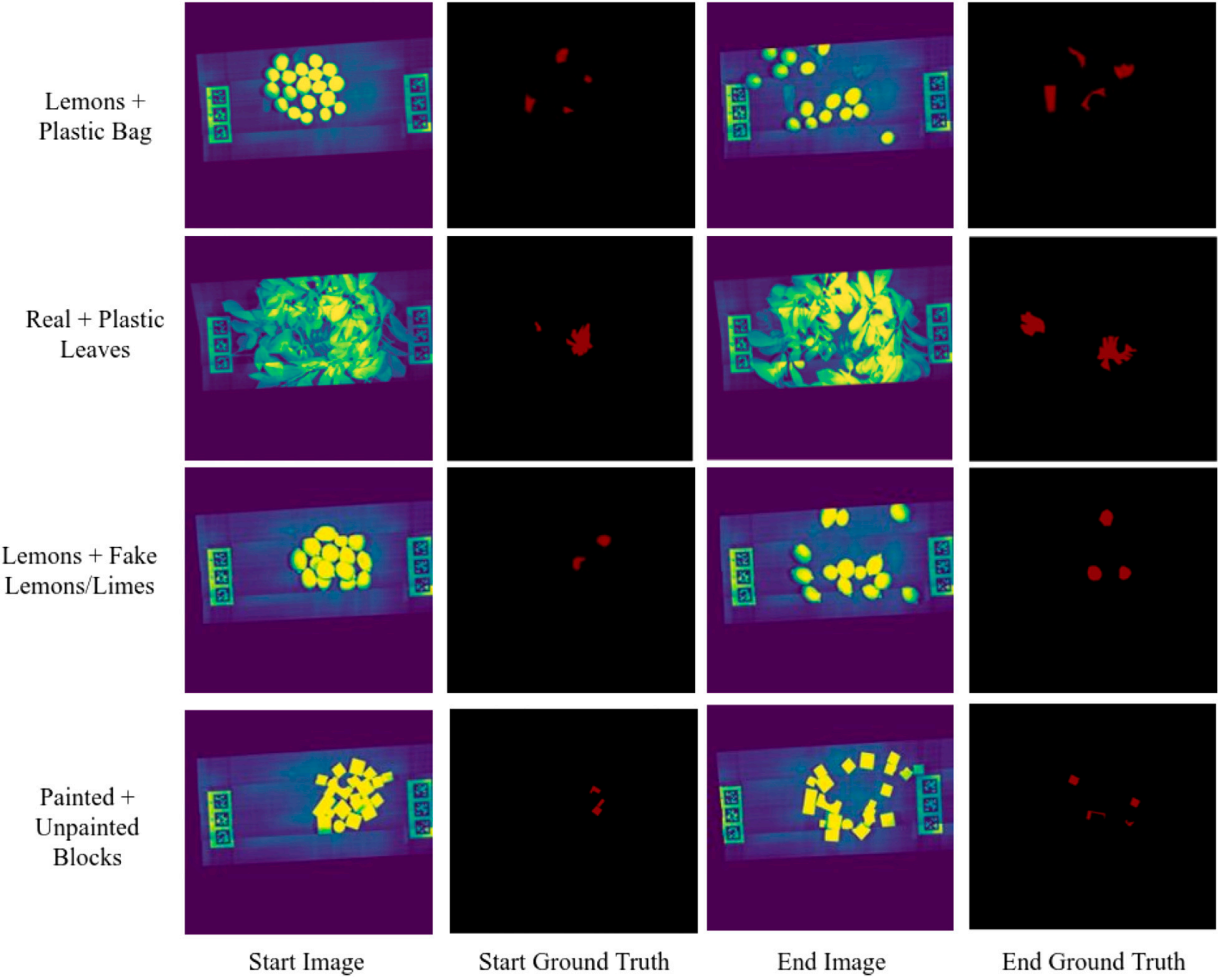
Four scenarios showing the pile of clutter after the initial scan, with associated ground truth labels for anomalous items. The end image shows the scene after disruption by three motion primitives, and ground truth shows the labeled anomalies in the scene.

Figure 10 shows an extracted wavelength channel from the associated, normalized datacube and the associated ground truth information.

### 8.2 Motion primitives

For deciding on actionable pixels, we note that single pixels are not strong indications of an anomaly; however, clusters of anomalous pixels within a region are a better marker. For this calculation, we turn to our previous superpixeling strategy to generate small pixel groups. Given a superpixel *p* containing normalized score values, the priority that the region should be perturbed with a pushing motion is given as:

$$\text{Priority}\left(p\right)=\left(\max\left(p\right)-\overset{∼}{p}\right)\sigma \left(p\right){\left(\overset{n}{\underset{i}{\sum }}p_{i}>\bar{p}\right)}^{2}$$

The notation $\overset{∼}{p}$ indicates the median value of the superpixel and $\bar{p}$ is its mean. We used median in contrast to mean since it avoided situations where a single erroneous pixel would skew the scores for the entire region. *σ*(*s*) designates the standard deviation of the superpixel. The calculation is designed to give maximum weight to regions with large standard deviations, indicating a relatively large reconstruction error, and multiple pixels that exceed the mean. The summation prevents sensor noise from the hyperspectral camera, occasionally manifesting in the form of dead pixels, from generating false positives. Our decision rule for detecting anomalous pixels is bound to an outlier detection approach when comparing the score of a single superpixel the collection of superpixels in the entire scene.

Following from the results of the anomaly detector and region prioritization, where the anomalies consistently identified, we utilized a per-pixel count for the true number of visible anomalies. By counting the number of regions identified as anomalous both before and after the application of pushing motions, we can quantify the tangible difference the arm made in exposing occluded clutter. Table 1 shows the results of the motion primitives and the percent change in the visible surface area of the exposed anomalies.

**TABLE 1:** Change in exposed anomalous pixels before and after application of pushing motions.

Scenario	Pre-motion pixels	Post-motion pixels	Change %
Lemons + Plastic Bags	576,308	1,222,574	112.14
Real + Plastic Leaves	881,866	2,009,668	127.89
Lemons + Fake Lemons/Limes	383,116	813,200	112.26
Painted + Unpainted Blocks	231,724	372,970	60.95

### 8.3 Receiver operator/precision-recall curve evaluation

**FIGURE 11:**
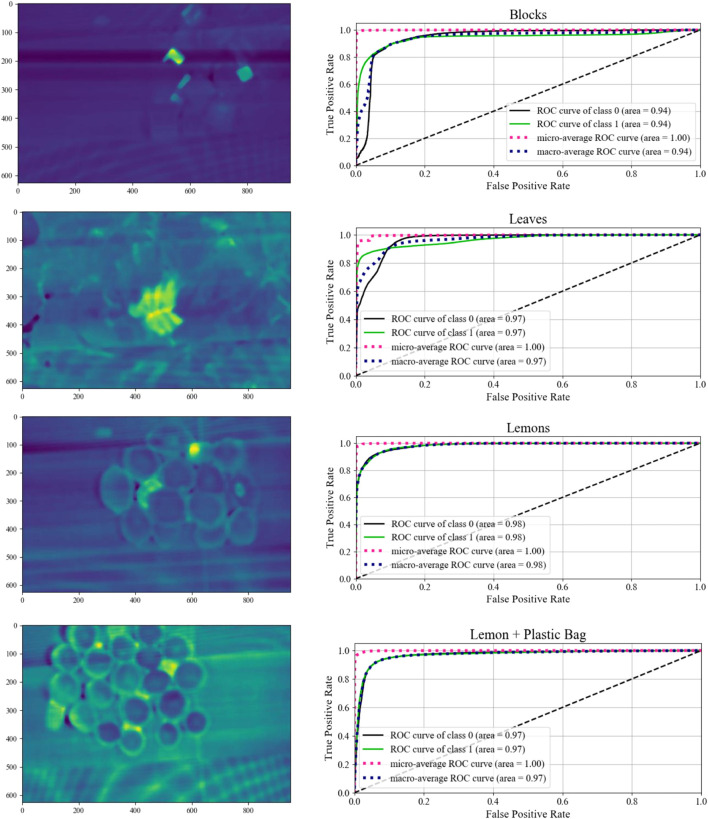
Mean absolute reconstruction error for each of the four environments and their associated Receiver Operator Curve (ROC) for multiple classification thresholds.

**FIGURE 12:**
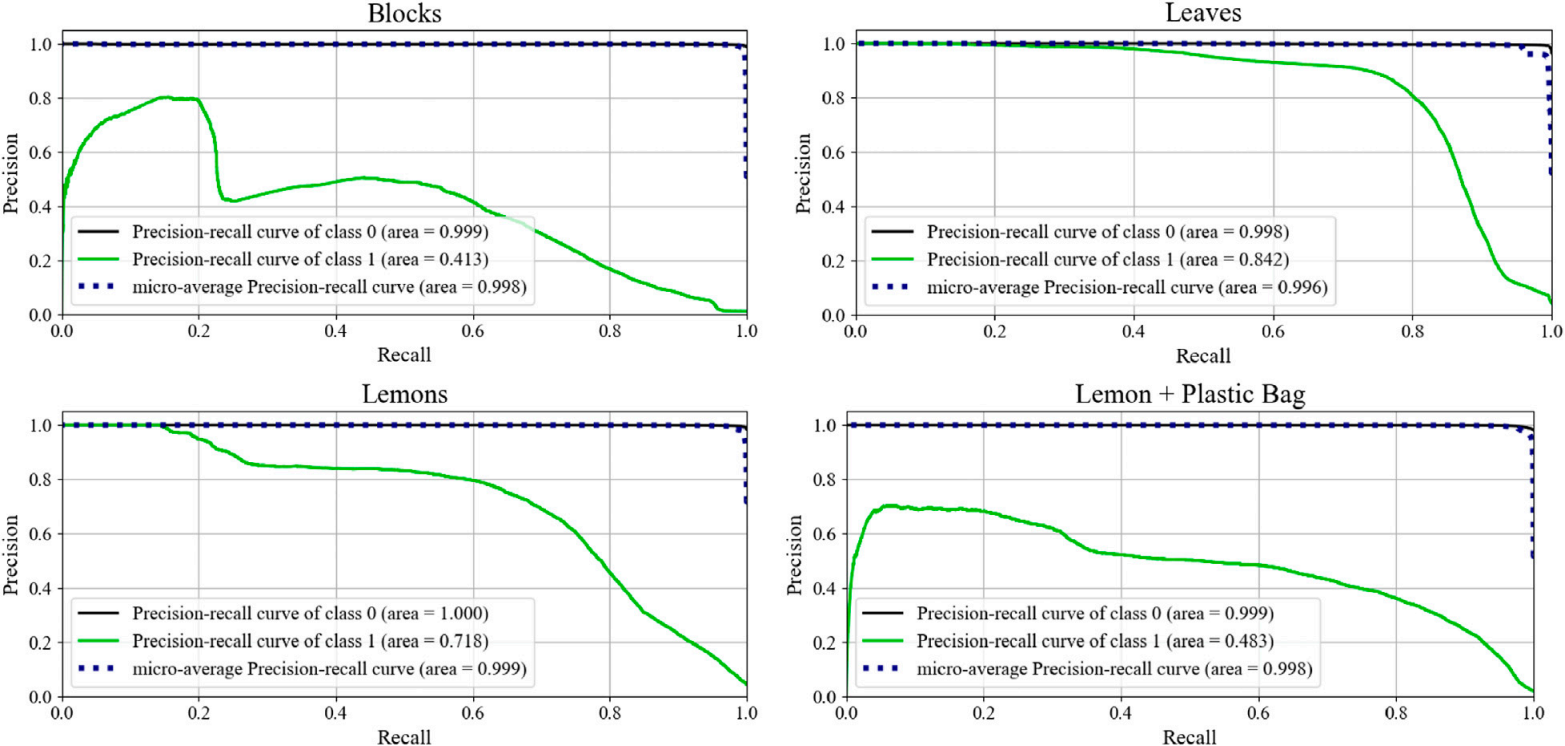
Precision recall curves for each of the scenarios. A consistently high precision score is desirable. A no-skill classifier will lie on the *x*-axis (recall).

As the anomaly detection algorithm is a variant of an unsupervised classification problem, we characterize our results with a Receiver Operator Curve (ROC) to evaluate the performance of the classifier as the classification threshold is altered. For each of the scenarios, we generate a plot showing the ROC curves as show in Figure 11. The ideal curve here would feature a curve immediately ascending to 1.0 for both the True Positive and False Positive rates, with an Area Under the Curve (AUC) of 1.0. In these graphics, class 0 is the pure material, and class 1 is the anomalous material. The ROC curve for the anomalies quickly ascends to a greater than 90% true positive rate before the false positive rate exceeds 10%. Along with the ROC curves, each scenario also includes an image presenting the reconstruction error for each pixel. The stronger yellow accents indicate a higher reconstruction error and a correspondingly higher probability of constituting an anomalous region Figure 12.

Although ROC curves are a classically used metric to demonstrate performance in hyperspectral anomaly detection, they can demonstrate skewed performance under situations of high class imbalance. The anomalies represent a fractional portion of the total information visible in the images. Under this imbalanced class distribution, the classifier performance is further evaluated by generating precision-recall (PR) curves. For both the leaves and the lemons, the anomaly classifier exemplifies strong performance as the recall increases. These results demonstrate that the model is able to consistently classify anomalous pixels, even those at lower threshold values. The Blocks and Lemon + Plastic Bag scenarios also demonstrate good initial performance, but then begins to fall off as recall increases. This initial spike indicate the highest probability points are correctly classified, while the lower thresholds are likely to be incorrectly classified. Although individual pixel values are important, averaging the probability across the superpixel has the two-fold effect of eliminating noise, and strengthening regions where a multi-pixel anomaly is present. Moreover, by design the robot is programmed to only act on the most probable regions to avoid unnecessary actions. The results in Section 8.2 further underscore the notion that good performance can be achieved by only executing a few motions on the most likely anomaly points. Table 2 directly compares the performance of both measures in each of the scenarios.

**TABLE 2:** Anomaly detector performance with the area under the Receiver Operator and Precision Recall Curves.

Scenario	AUC ROC	AUC PR
Lemons + Plastic Bags	0.97	0.483
Real + Plastic Leaves	0.97	0.842
Lemons + Fake Lemons/Limes	0.98	0.718
Painted + Unpainted Blocks	0.94	0.413

### 8.4 Ablation study

**FIGURE 13:**
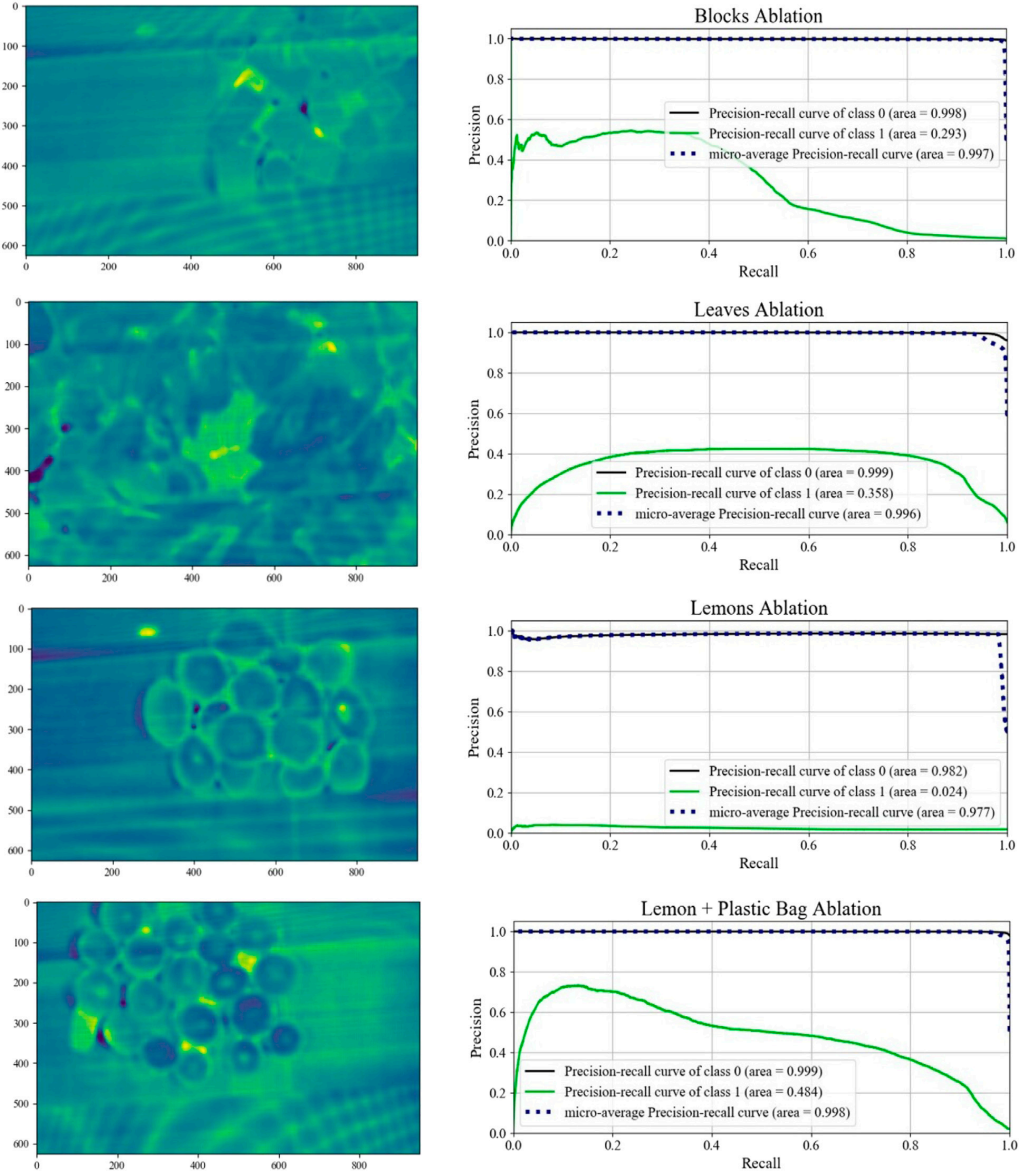
Mean absolute reconstruction error for each of the four environments and their associated precision recall curves conducted for the ablation study.

As a further validation of our two stage anomaly detector, we remove the first stage of the pipeline to avoid any of the initial clustering; excluding superpixeling, noise adjusted PCA, and *k*-means segmentation. In this scenario, the autoencoder is trained on the entirety of the registered hyperspectral datacube obtained directly after data registration 4. As a consequence, the autoencoder is given training samples for each of the anomalous pixels, and attempts to reconstruct the sample spectra while minimizing the MSE. Figure 13 shows the results of removing the data segmentation from the pipeline. As before, yellow indicates the strong likelihood of a pixel being anomalous. Comparing the results excluding segmentation to those which include segmentation in Figure 11, we see that the non-segmented results are in general noisier. The presence of darker violet indicates a comparatively low reconstruction error. In particular the block, leaves, and lemons environments are all substantially noisier when not segmented as compared to the pipeline which includes segmentation. This observation is also reflected in the ablation precision-recall curves. Each of these three reduced scenarios has a smaller AUC than the pipeline which includes segmentation. This result corresponds to worse classifier performance at every possible decision threshold. From these results, the clustering step functions sufficiently as a noise removal step, allowing the autoencoder to learn a better representation of the dominant class. In the ablation study, the presence of anomalies increases the average noise, even if the anomalies trigger a slightly stronger response than the average pixel.

## 9 Discussion

The results presented above demonstrate the effectiveness of the system by quickly identifying anomalies without operator intervention. We note that on rare occurrences, a poor initialization of the center of the *k*-means clusters could lead to poor initial segmentation of the trainable data. This segmentation was generally over-conservative, and excludes additional pure regions that ideally could contribute to the model training. Even in the midst of such poor initializations, the autoencoder was able to learn a generally good representation of the non-anomalous class, although the reconstruction error in such situations was generally limited.

In the results for the anomaly detector, we see surprisingly good performance of the detector in identifying other anomalies not considered part of the original clutter. For instance, in the lemons environment, the scan not only detected the partially occluded lemons, but one lemon which had a grocery store sticker on it was also flagged as highly anomalous. Additionally, some of the leaves are connected to woody stems, which peek through the rest of the leaves. Although we did not consider stems as initially part of the anomaly class, their minimal presence and spectral difference from leaves with an abundance of chlorophyll logically follows.

The motion primitives demonstrate impressive improvements in exposing object clutter. In 3 of the 4 scenarios, impurity surface area was increased by over 100%, and in the other scenario (painted blocks) the change in surface area was non-negligible. The approach proved to be generalizable to multiple scenarios and fast to execute. Planning from the time of identification of the target coordinate, to optimization of the pushing trajectory averaged 7 s to generate the joint angles needed to achieve the desired motion. As it takes several minutes to acquire a hyperspectral datacube with a pushbroom camera, we reuse the same hyperspectral data in the determination of target regions of interest for all motion primitives. This approach succeeds with items that will not move beyond the point of applied motion, but can cause discrepancies with objects that continue to roll, such as lemons and impact objects not in the gripper path of motion. Pushing also had the effect of creating a greater distribution of the scene anomalies, creating better opportunities for grasping. In Figure 10, the end ground truth images demonstrate a greater distribution of anomalies over the image plane. A wider spread is advantageous for grasp planning, as isolation of the anomalies from the pure items allows the target item to be cleanly grasped with minimized risk of perturbing unnecessary items.

In the ablation study, the lemon + plastic bag scenario generates similar results to the two stage pipeline. The AUC in both setups is also similar. This could be a result of the plastic bag pieces being different in both color and material, leading to a greater difference in the mean spectral signatures of the two items. Nonetheless, this observation shows that the two-stage pipeline only improves results, and is able to identify anomalies that are visually similar, which supports the generality of this work.

In future work, we will study the selection of motion primitives and how they are heuristically generated. The formulation we employed in the current scope of work made assumptions regarding the nature of clutter. Specifically, we assumed the objects would be separable with linear motions in the x-y plane. However as the leaf example best demonstrated, the presence of items with large surface areas and contact between items causes difficulty in separation. This fact is further exacerbated when the coefficient of friction is lower between the table and the items than between items. Initial pushing and nudging could be used to create larger visibility of previously occluded items. With an opening into the clutter at least as large as the gripper finger width, the items could then be grasped and removed. Additionally, the UR3e co-robot used in this research has a limited working distance. This constraint limited the number of cluttered points that could be properly addressed to within an area generally beneath the robot. Increasing the size of the robot should ameliorate this concern and enable motion planning to all points in the visible scene. Improving the diversity of motions available to declutter the workspace will increase the versatility and generality of the proposed algorithm.

## 10 Conclusion

In this paper, we present a novel generalizable model to detect and expose partially occluded anomalies in a cluttered scene. Our model does not assume prior semantic knowledge of the scene or the objects contained within, operating on the assumption that the anomalies are the minority of objects in the scene. Our methodology circumvents the intensive process of needing to curate labels for comparable problems solved with purely RGB data. We accomplish this by registering together hyperspectral and RGB-Depth data to create an information rich model of the environment which can be interpreted with magnitudes greater granularity than RGB data. Through feeding an AE definitively pure regions from the registered HSI-RGB-Depth data selected with iterative clustering, we train a more complex generalizable model where anomalies are detected in regions with high reconstructive loss. We found that traditional approaches to detecting anomalies within hyperspectral images either do not work for regions where anomaly sizes are greater than a few pixels, or are prohibitively expensive computationally, taking far too long to run. We found that our approach works well in situations where purely RGB-driven solutions likely would fail, as demonstrated in our experiments where we are able to successfully differentiate plastic fruit from their visually similar real fruit counterparts. With the information obtained from our anomaly detection AE, we are able to uncover potentially occluded anomalies by selecting an optimal plan from our heuristic motion primitives.

Moving forwards, we plan to increase the robustness and speed of our system, as well as to decrease the collection period of the scans. Our pushbroom hyperspectral camera requires several passes to survey a workspace, passes that could be eliminated entirely by a snapshot hyperspectral camera with a reasonable horizontal field of view. We plan to increase the robustness and speed of the scans by optimizing or scheduling our algorithms involved with registering data and creating the pure feed for the AE. This work represents a substantial step towards meaningful integration of robot-centric hyperspectral data into the handling of cluttered environments.

## Data Availability

The raw data supporting the conclusion of this article will be made available by the authors, without undue reservation.

## References

[B1] Achanta R. Shaji A. Smith K. Lucchi A. Fua P. Süsstrunk S. (2010). Slic superpixels. Tech. rep. 10.1109/TPAMI.2012.120 22641706

[B2] Arisoy S. Nasrabadi N. M. Kayabol K. (2021). Unsupervised pixel-wise hyperspectral anomaly detection via autoencoding adversarial networks. IEEE Geosci. Remote Sens. Lett. 19, 1–5. 10.1109/lgrs.2021.3049711

[B3] Barnabé P. Dislaire G. Leroy S. Pirard E. (2015). Design and calibration of a two-camera (visible to near-infrared and short-wave infrared) hyperspectral acquisition system for the characterization of metallic alloys from the recycling industry. J. Electron. Imaging 24, 061115. 10.1117/1.jei.24.6.061115

[B4] Bejjani W. Dogar M. R. Leonetti M. (2019). “Learning physics-based manipulation in clutter: Combining image-based generalization and look-ahead planning,” in 2019 IEEE/RSJ International Conference on Intelligent Robots and Systems (IROS) (IEEE), 6562–6569.

[B5] Bruegge C. J. Stiegman A. E. Rainen R. A. Springsteen A. W. (1993). Use of spectralon as a diffuse reflectance standard for in-flight calibration of earth-orbiting sensors. Opt. Eng. 32, 805–814. 10.1117/12.132373

[B6] Buades A. Coll B. Morel J.-M. (2011). Non-local means denoising. Image Process. Line 1, 208–212. 10.5201/ipol.2011.bcm_nlm

[B7] Chen B. Shi S. Sun J. Gong W. Yang J. Du L. (2019). Hyperspectral lidar point cloud segmentation based on geometric and spectral information. Opt. Express 27, 24043. 10.1364/OE.27.024043 31510299

[B8] Debes C. Merentitis A. Heremans R. Hahn J. Frangiadakis N. van Kasteren T. (2014). Hyperspectral and lidar data fusion: Outcome of the 2013 grss data fusion contest. IEEE J. Sel. Top. Appl. Earth Obs. Remote Sens. 7, 2405–2418. 10.1109/jstars.2014.2305441

[B9] Erickson Z. Luskey N. Chernova S. Kemp C. C. (2019). Classification of household materials via spectroscopy. IEEE Robot. Autom. Lett. 4, 700–707. 10.1109/lra.2019.2892593

[B10] Erickson Z. Xing E. Srirangam B. Chernova S. Kemp C. C. (2020). “Multimodal material classification for robots using spectroscopy and high resolution texture imaging,” in 2020 IEEE/RSJ International Conference on Intelligent Robots and Systems (IROS) (IEEE), 10452–10459.

[B11] Ester M. Kriegel H.-P. Sander J. Xu X. (1996). “A density-based algorithm for discovering clusters in large spatial databases with noise,” in KDD-96 Proceedings, 226–231.

[B12] Fischler M. A. Bolles R. C. (1981). Random sample consensus: a paradigm for model fitting with applications to image analysis and automated cartography. Commun. ACM 24, 381–395. 10.1145/358669.358692

[B13] Foote T. (2013). “tf: The transform library,” in 2013 IEEE Conference on Technologies for Practical Robot Applications (TePRA) (IEEE), 1–6.

[B14] Hanson N. Hochsztein H. Vaidya A. Dorsey K. Padir T. (2022a). “In-hand object recognition with innervated fiber optic spectroscopy for soft grippers,” in 2022 5th IEEE International Conference on Soft Robotics, RoboSoft 2022 (IEEE).

[B15] Hanson N. Kelestemur T. Berman J. Ritzenhoff D. Padir T. (2022). “Hyperbot – A benchmarking testbed for acquisition of robot-centric hyperspectral scene and in-hand object data,” in 2022 12th Workshop on Hyperspectral Imaging and Signal Processing: Evolution in Remote Sensing (WHISPERS) (IEEE).

[B16] Hanson N. Kelestemur T. Erdogmus D. Padir T. (2022b). Pregrasp object material classification by a novel gripper design with integrated spectroscopy. [Dataset]. 10.48550/ARXIV.2207.00942

[B17] Hanson N. Shaham M. Erdogmus D. Padir T. (2022c). “Vast: Visual and spectral terrain classification in unstructured multi-class environments,” in 2022 IEEE/RSJ International Conference on Intelligent Robots and Systems (IROS) (IEEE). To appear.

[B18] Khodadadzadeh M. Li J. Prasad S. Plaza A. (2015). Fusion of hyperspectral and lidar remote sensing data using multiple feature learning. IEEE J. Sel. Top. Appl. Earth Obs. Remote Sens. 8, 2971–2983. 10.1109/jstars.2015.2432037

[B19] Ku Y. Yang J. Fang H. Xiao W. Zhuang J. (2021). Deep learning of grasping detection for a robot used in sorting construction and demolition waste. J. Mater. Cycles Waste Manag. 23, 84–95. 10.1007/s10163-020-01098-z

[B20] Kurillo G. Hemingway E. Cheng M.-L. Cheng L. (2022). Evaluating the accuracy of the azure kinect and kinect v2. Sensors 22, 2469. 10.3390/s22072469 35408082 PMC9002889

[B21] Kwak D.-H. Son G.-J. Park M.-K. Kim Y.-D. (2021). Rapid foreign object detection system on seaweed using vnir hyperspectral imaging. Sensors 21, 5279. 10.3390/s21165279 34450722 PMC8400334

[B22] Lee J. Woodyatt A. Berman M. (1990). Enhancement of high spectral resolution remote-sensing data by a noise-adjusted principal components transform. IEEE Trans. Geosci. Remote Sens. 28, 295–304. 10.1109/36.54356

[B23] Levenberg K. (1944). A method for the solution of certain non-linear problems in least squares. Q. Appl. Math. 2, 164–168. 10.1090/qam/10666

[B24] Li W. Du Q. (2015). Collaborative representation for hyperspectral anomaly detection. IEEE Trans. Geosci. Remote Sens. 53, 1463–1474. 10.1109/TGRS.2014.2343955

[B25] Lowe D. G. (2004). Distinctive image features from scale-invariant keypoints. Int. J. Comput. Vis. 60, 91–110. 10.1023/b:visi.0000029664.99615.94

[B26] Ma N. Peng Y. Wang S. Leong P. (2018). An unsupervised deep hyperspectral anomaly detector. Sensors 18, 693. 10.3390/s18030693 PMC5877305 29495410

[B27] Mao S. Ji M. Wang B. Dai Q. Fang L. (2022). Surface material perception through multimodal learning. IEEE J. Sel. Top. Signal Process. 16, 843–853. 10.1109/jstsp.2022.3171682

[B28] Matteoli S. Diani M. Corsini G. (2010). A tutorial overview of anomaly detection in hyperspectral images. IEEE Aerosp. Electron. Syst. Mag. 25, 5–28. 10.1109/maes.2010.5546306

[B29] Muja M. Lowe D. G. (2014). Scalable nearest neighbor algorithms for high dimensional data. IEEE Trans. Pattern Anal. Mach. Intell. 36, 2227–2240. 10.1109/TPAMI.2014.2321376 26353063

[B30] Nair V. Hinton G. E. (2010). “Rectified linear units improve restricted Boltzmann machines,” in Proceedings of the 27th International Conference on Machine Learning (ICML-10).

[B31] Quigley M. Conley K. Gerkey B. Faust J. Foote T. Leibs J. (2009). “Ros: an open-source robot operating system,” in ICRA workshop on open source software, Kobe, Japan, 5.

[B32] Rasti B. Ghamisi P. Gloaguen R. (2017). Hyperspectral and lidar fusion using extinction profiles and total variation component analysis. IEEE Trans. Geosci. Remote Sens. 55, 3997–4007. 10.1109/tgrs.2017.2686450

[B33] Reed I. Yu X. (1990). Adaptive multiple-band cfar detection of an optical pattern with unknown spectral distribution. IEEE Trans. Acoust. 38, 1760–1770. 10.1109/29.60107

[B34] Rublee E. Rabaud V. Konolige K. Bradski G. (2011). “Orb: An efficient alternative to sift or surf,” in 2011 International conference on computer vision (IEEE), 2564–2571.

[B35] Shahinfar S. Meek P. Falzon G. (2020). How many images do i need?” understanding how sample size per class affects deep learning model performance metrics for balanced designs in autonomous wildlife monitoring. Ecol. Inf. 57, 101085. 10.1016/j.ecoinf.2020.101085

[B36] Shorten C. Khoshgoftaar T. M. (2019). A survey on image data augmentation for deep learning. J. Big Data 6, 60–48. 10.1186/s40537-019-0197-0 PMC8287113 34306963

[B37] Su H. Wu Z. Zhang H. Du Q. (2022). Hyperspectral anomaly detection: A survey. IEEE Geosci. Remote Sens. Mag. 10, 64–90. 10.1109/MGRS.2021.3105440

[B38] Taitano Y. P. Geier B. A. Bauer K. W. Jr (2010). A locally adaptable iterative rx detector. EURASIP J. Adv. Signal Process. 2010, 341908. 10.1155/2010/341908

[B39] Thananjeyan B. Kerr J. Huang H. Gonzalez J. E. Goldberg K. (2022). All you need is luv: Unsupervised collection of labeled images using invisible uv fluorescent indicators. [Dataset]. 10.48550/ARXIV.2203.04566

[B40] Vali A. Krämer P. Strzoda R. Comai S. Gigler A. M. Matteucci M. (2021). “Hyperspectral image analysis for automatic detection and discrimination of residual manufacturing contaminants,” in 2021 26th IEEE International Conference on Emerging Technologies and Factory Automation (ETFA) (IEEE), 1–8.

[B41] Wang J. Olson E. (2016). “Apriltag 2: Efficient and robust fiducial detection,” in 2016 IEEE/RSJ International Conference on Intelligent Robots and Systems (IROS) (IEEE), 4193–4198.

[B42] Weik M. H. (2001). “Spectral width,” in Computer science and communications dictionary (Boston, MA: Springer US), 1633.

[B43] Xu Y. Zhang L. Du B. Zhang L. (2022). Hyperspectral anomaly detection based on machine learning: An overview. IEEE J. Sel. Top. Appl. Earth Obs. Remote Sens. 15, 3351–3364. 10.1109/jstars.2022.3167830

[B44] Zeng A. Song S. Yu K.-T. Donlon E. Hogan F. R. Bauza M. (2018a). “Robotic pick-and-place of novel objects in clutter with multi-affordance grasping and cross-domain image matching,” in 2018 IEEE international conference on robotics and automation (ICRA) (IEEE), 3750–3757.

[B45] Zeng Z. Zhou Z. Sui Z. Jenkins O. C. (2018b). “Semantic robot programming for goal-directed manipulation in cluttered scenes,” in 2018 IEEE international conference on robotics and automation (ICRA) (IEEE), 7462–7469.

[B46] Zhang R. Xu L. Yu Z. Shi Y. Mu C. Xu M. (2021). Deep-irtarget: An automatic target detector in infrared imagery using dual-domain feature extraction and allocation. IEEE Trans. Multimed. 24, 1735–1749. 10.1109/tmm.2021.3070138

[B47] Zhao C. Wang Y. Qi B. Wang J. (2015). Global and local real-time anomaly detectors for hyperspectral remote sensing imagery. Remote Sens. 7, 3966–3985. 10.3390/rs70403966

[B48] Zuiderveld K. (1994). “Contrast limited adaptive histogram equalization,” in Graph. gems. Academic Press Professional, Inc., 474–485. 10.1016/b978-0-12-336156-1.50061-6

